# Contributing factors to influenza vaccine uptake in general hospitals: an explorative management questionnaire study from the Netherlands

**DOI:** 10.1186/1471-2458-12-1101

**Published:** 2012-12-21

**Authors:** Josien Riphagen-Dalhuisen, Joep CF Kuiphuis, Arjen R Procé, Willem Luytjes, Maarten J Postma, Eelko Hak

**Affiliations:** 1 Department of Pharmacy, Division of Pharmacoepidemiology and Pharmacoeconomics, University of Groningen, Antonius Deusinglaan 1, P.O. Box XB45, 9713 AV, Groningen, the Netherlands; 2 Department of Epidemiology, University Medical Centre Groningen, P.O. Box 30.001, 9700 RB, Groningen, the Netherlands; 3 Dutch Vaccine Institute (NVI)/National Institute for Public Health and the Environment (RIVM), P.O. Box 1, 3720 BA, Bilthoven, the Netherlands

**Keywords:** Health care workers, Influenza vaccination, General hospital, Management

## Abstract

**Background:**

The influenza vaccination rate in hospitals among health care workers in Europe remains low. As there is a lack of research about management factors we assessed factors reported by administrators of general hospitals that are associated with the influenza vaccine uptake among health care workers.

**Methods:**

All 81 general hospitals in the Netherlands were approached to participate in a self-administered questionnaire study. The questionnaire was directed at the hospital administrators. The following factors were addressed: beliefs about the effectiveness of the influenza vaccine, whether the hospital had a written policy on influenza vaccination and how the hospital informed their staff about influenza vaccination. The questionnaire also included questions about mandatory vaccination, whether it was free of charge and how delivered as well as the vaccination campaign costs. The outcome of this one-season survey is the self-reported overall influenza vaccination rate of health care workers.

**Results:**

In all, 79 of 81 hospitals that were approached were willing to participate and therefore received a questionnaire. Of these, 42 were returned (response rate 52%). Overall influenza vaccination rate among health care workers in our sample was 17.7% (95% confidence interval: 14.6% to 20.8%). Hospitals in which the administrators agreed with positive statements concerning the influenza vaccination had a slightly higher, but non-significant, vaccine uptake. There was a 9% higher vaccine uptake in hospitals that spent more than €1250,- on the vaccination campaign (24.0% versus 15.0%; 95% confidence interval from 0.7% to 17.3%).

**Conclusions:**

Agreement with positive statements about management factors with regard to influenza vaccination were not associated with the uptake. More economic investments were related with a higher vaccine uptake; the reasons for this should be explored further.

## Background

A large number of studies from different regions and among different healthy adult populations have demonstrated that seasonal influenza vaccination is effective in preventing influenza infection [1-5]. In acute health care settings it is essential to protect patients against influenza because most of them are vulnerable at admission for infections and its complications. Because of person-to-person transmission and intensive contacts with patients, vaccination of health care workers has been suggested to indirectly benefit patients [6]. There is also some evidence that vaccinating health care workers against influenza reduces costs in health care by reducing the length of hospitalization and reducing absenteeism of health care workers, though some did not find an effect on absenteeism rates [1,7,8]. Lastly, there are ethical arguments in favor of vaccination, like health care workers’ primary duty not to harm their patients.

Despite the potential benefits of vaccination, its uptake in hospitals among health care workers in Europe remains low. In 2003 Kroneman et al. showed vaccine uptake rates among health care workers of five European countries ranging from 15% in the UK and Germany to 25% in Romania [9]. More recently, the survey of Blank et al. also demonstrated low overall influenza vaccine coverage rates among health care workers in eleven European countries which ranged from 6.4% in Poland to 26.3% in Czech Republic in the 2007/2008 influenza season [10]. Vaccination rates exceeding 50% are difficult to reach [11,12].

To improve vaccine uptake, several behavioral factors are essential to be targeted and different methods should be applied to increase vaccine uptake [13]. For example, in most studies a positive relation with knowledge about the vaccine’s efficacy and side effects and the importance not to harm patients is found. Several interventions targeting these determinants can influence the uptake such as educational materials, interactive sessions, role models, facilitating access like the use of mobile carts and the dedication of a person to coordinate the campaign. Some hospitals in the Netherlands have already implemented a vaccination campaign, but the relevant management factors have been under-explored in the worldwide literature. In this study a questionnaire was used to assess and quantify the factors reported by administrators of the general hospitals in the Netherlands regarding influenza vaccine uptake among health care workers.

## Methods

All 81 Dutch general hospitals were approached for this study in December 2010. University hospitals were excluded because there was already an intervention program implemented in these hospitals as part of an ongoing trial [registration no. NCT01481467]. These 81 hospitals were contacted by telephone for participation and 79 out of 81 hospitals were willing to participate. The questionnaire was sent on December 6^th^ 2010 to the participating 79 hospitals and, if necessary, after two weeks a reminder was sent. In the beginning/mid January the hospital managements that did not return the questionnaire were contacted again by telephone as a reminder.

The hospitals in the Netherlands are all publicly funded, not private nor specialty clinics, and we did not contact university medical centers, since they were part of a trial on influenza vaccination uptake. In the Netherlands all persons with risk-elevating conditions can get the vaccine via their general practitioner. Among HCWs this proportion is less than 5% [6].

The following items were assessed in the self-administered questionnaire: the overall influenza vaccination rate of health care workers in the hospital, the opinion about the effectiveness of the influenza vaccine, whether the hospital had a written policy on influenza vaccination and how the hospital informed their staff about influenza vaccine, e.g. personal by mail or letter, through general written information by posters or the intranet, or in the form of group meetings. The questionnaire also included questions about mandatory vaccination and, whether it was free of charge and how it was organized and about the program costs.

The study was part of a trial [registration no. NCT01481467] and the protocol of the trial was waived by the medical ethical committee of the University Medical Center Groningen for ethical approval according to the Dutch Law of Research with Humans (No. 2009.267). The study was conducted in accordance with the Dutch Law for the Protection of Personal Data (Wet Bescherming Persoonsgegevens) and the Declaration of Helsinki [http://www.wma.net/e/policy/b3.htm].

### Statistical analysis

Data were analyzed using SPSS version 18.0. To determine which predictors were associated with mean influenza vaccination rates independent t-tests were used. 95% confidence intervals (95% CI) were calculated to determine statistical significance at a p-level of 5%.

## Results

A questionnaire was sent to 79 of a total of 81 hospitals. Eventually, the questionnaire was returned by 42 hospitals (52% response rate). The size of the hospitals ranged from 600 to 5,500 health care workers. The average vaccination rate for influenza in this sample was 17.7% (median value 16.0%, minimum 0.5% and maximum 45.4%, 95% CI 14.6% to 20.8%).

Health care workers were invited for influenza vaccination personally by mail in 26% of hospitals, and 100% used general written information for all health care workers. Only 3% organized information meetings about influenza vaccination. In all, 100% of the hospitals supplied their health care workers with influenza vaccination free of charge. Vaccines were administered at the departments in 58% of hospitals, 84% had mobile carts, 97% had a central location to administer vaccines and only 4% vaccinated at special request.

As shown in Table 1, the majority of management of hospitals agreed with the first three items (vaccination effects mortality and both health care workers and hospital managements have a special responsibility in protecting patients and offering vaccination). Thirty of the 42 hospital administrators (71.4%) believed that vaccinating against influenza has an effect on mortality of patients in the hospital. However, when vaccination rates remain too low only three hospitals (7.1%) would consider implementing a mandatory vaccination program.

**Table 1:** Agreement of hospital management on questions concerning influenza vaccination (N=42)

**Question/statement**	**Management of hospital that agrees, N (%)**
Vaccinating against influenza has effect on mortality of patients in the hospital.	30 (71.4)
Health care workers with patient contact have a special responsibility in preventing infection of their patients.	38 (90.5)
The management of the hospital has a moral responsibility of offering influenza vaccination to their health care workers.	35 (83.3)
An intervention program with the purpose to stimulate vaccination has a positive effect on vaccination rate.	19 (45.2)
The management of the hospital would implement such an intervention program to raise vaccination rate.	22 (52.3)
The management of the hospital considers mandatory vaccination when vaccination rate remains too low.	3 (7.1)
A mandatory vaccination against influenza will reduce costs in the hospital.	12 (28.6)
The vaccine against influenza is effective.	29 (69.0)

Half of the hospital managements thought that an intervention program could raise the vaccination rate. Further, 19 administrators (45.2%) believed that an intervention program would have a positive effect on vaccination rate. Management of 29 hospitals (69.0%) believed that the vaccine is effective against influenza.

In Table 2 is shown how the factors were related to the average vaccination rate. When health care workers are personally informed about influenza vaccination, the average vaccination rate is somewhat higher than any other form of providing information (18.9% compared to 15.6%, 95%CI −2.97% to 9.70%). The managements’ positive beliefs about the effect of vaccination on mortality of patients was associated with an average vaccination rate of 19.0% compared to 16.7% when there were negative beliefs about this effect.

**Table 2:** Agreement of management of hospitals (N=42) with possible predictors of vaccination rate and mean vaccination rate

**Predictor**	**Agreement Yes N (%)**	**Agreement No N (%)**	**Mean difference (95% CI)**
Health care workers are personally informed about influenza vaccination	24/38 (18.9)	14/38 (15.6)	3.36 (−2.97 to 9.70)
Agreement with the effect of vaccination on mortality of patients	27/33 (19.0)	6/33 (16.7)	2.24 (−6.50 to 10.98)
Agreement of management with the statement that they are responsible for offering the vaccine to health care workers	32/35 (18.8)	3/35 (10.0)	8.78 (−2.75 to 20.32)
Believing that an intervention program to stimulate vaccination has a positive effect on vaccination rate	18/26 (16.5)	8/26 (17.3)	−0.85 (−8.15 to 6.46)
Hospitals willing to implement an intervention program	20/25 (17.4)	5/25 (12.7)	4.70 (−2.66 to 12.06)
Hospitals willing to implement mandatory vaccination	3/33 (18.0)	30/33 (17.5)	0.51 (−11.49 to 12.51)
Believing that mandatory vaccination will reduce costs	11/24 (16.7)	13/24 (15.6)	1.08 (−5.23 to 7.38)
Believing that the vaccine against influenza is effective	27/32 (18.7)	5/32 (14.2)	4.48 (−5.20 to 14.16)

(In brackets: mean % of influenza vaccine uptake among health care workers).

In hospitals where management agreed to be responsible for offering the vaccine to health care workers an average vaccination rate of 18.8% was observed opposed to 10.0% in hospitals in which management disagreed with being responsible.

In all, 11 out of 42 hospital management believed mandatory vaccination will reduce costs. Of these hospitals, the ones that agreed had an average vaccination rate of 16.7% and the ones that disagreed had an average vaccination rate of 15.6%. When asked if they wanted to implement a mandatory vaccination only three hospitals were willing to do so.

The costs of the annual flu campaign and the actual vaccination differed a lot between general hospitals. The average costs for the annual influenza vaccination campaign in 2010 were €640.38 per hospital with a minimum of €0.00 and a maximum of €2000.00 (standard deviation 563.21). The average costs for vaccination were €4198.54 per hospital with a minimum of €0.00 and a maximum of €14262.50 (standard deviation 3643.61).

**Figure 1:**
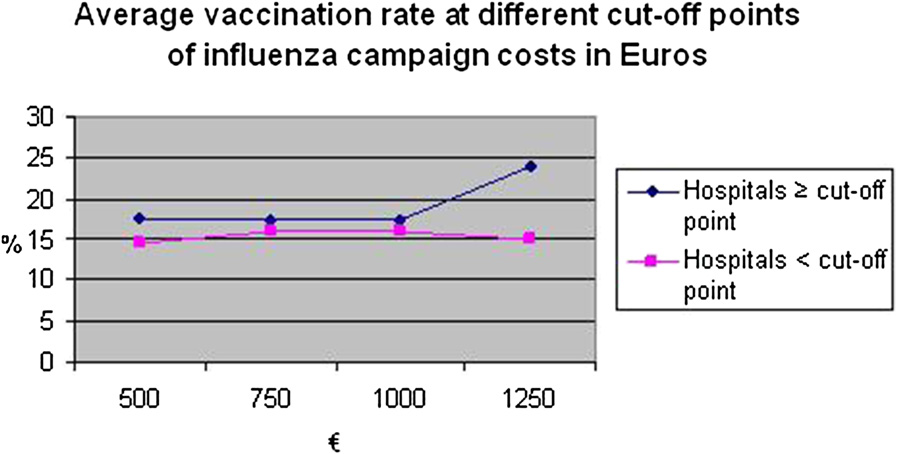
**Average vaccination rate at different cut-off points of influenza campaign costs in Euros.**N=25 (vaccination rate in %).

In Figure 1 the costs of the vaccination campaigns are compared to the vaccination rate, showing a higher vaccine uptake among HCWs in hospitals which spent more money on their vaccination campaign. To assess if a more expensive influenza campaign is correlated with a higher vaccination rate an independent t-test was performed. Only four hospitals spent more than €1250 on the influenza campaign. The average vaccination rate of these hospitals was 24.0% compared to 15.0% of hospitals that spent less than €1250 (mean difference 8.97; p<0.05), demonstrating a higher vaccine uptake among HCWs in hospitals which spent more than €1250 on their vaccination campaign. These differences remained if analyzed according to size of the hospital (25% versus 18% in hospitals with less than 2,000 health care workers and 23% and 14% in hospitals with more than 2000 health care workers).

## Discussion

In this study we found that agreement of hospital management with positive statements about influenza vaccination was not associated with influenza vaccine uptake. The average influenza vaccination rate among health care workers in our sample of Dutch hospitals was low; less than one in five received the vaccine. However, this is similar to the European situation [9-11]. In theory, one would expect that health care workers that are better informed about influenza vaccination, e.g. by personal information, have a higher vaccination rate because of a better understanding of the need to be vaccinated. As can be seen in Table 2 there is no significant difference in mean vaccine uptake between hospitals that personally inform their health care workers and hospitals that do not. This could be explained by the fact that hospitals invest only marginal in informing their health care workers in the proper way or they fail to deliver the personal messages to their staff.

The total response rate in the general hospitals was 52% which is quite high for a questionnaire study. However, response bias might have influenced the results. Since it is unknown what the actual current characteristics are of the non-responder hospitals, we were not able to compare them with the responders. We do believe however that the potential for selection bias is not large and more depending on the time and availability of the contact person (which is highly unlikely to be associated with the type of hospital). Importantly, there was a large variation in size of hospitals and agreements with statements, hence the associations between factors and vaccine uptake are most likely not influenced by this type of bias. Also, the average vaccination rate in our sample could not be weighted by the size of the hospitals to obtain a national estimate, so the 17% as observed in this study should not be directly accepted as a national estimate. However, as mentioned above, the sample may be assumed as rather representative of the total hospital population. Further, we asked about the percentage of health care workers being vaccinated and did not actually count vaccinees and total number of health care workers. Since it is important for quality management and for financial reasons, most hospitals do have accurate figures on this preventive method. In addition, another limitation of this study is that we have not taken into account other potential confounders in our analyses, like age structure of the hospital and hospital size. Lastly, it is unknown how many HCWs in these hospitals were already vaccinated against influenza by their general practitioner.

Most of the factors contributing to a slightly higher vaccination rate were only marginally related to a higher vaccine uptake. The questionnaires were directed at management of the hospital – for this reason the statements are the statements of the management and not necessarily of the whole hospital. Although in general it appeared that the studied beliefs of the administrators were not essential in raising the vaccine uptake, it may be that there are elements of these beliefs that may well be important. Detailed factors on how exactly HCWs were informed or motivated for vaccination could be of relevance and we therefore would advocate to study these in more detail using qualitative techniques such as focus groups in addition to what we already know from questionnaire studies [14]*.* The difference in vaccination costs can be explained by the fact that some hospitals have more health care workers than others. The correlation between investing in educational campaigns apparently leads to higher vaccination rates, even if results were obtained from small or larger hospitals. Therefore, when hospitals invest in educational materials to inform their health care workers that vaccination against influenza will protect their patients, vaccination rates are expected to be higher.

The fact that 11 hospitals think mandatory vaccination reduces costs but only three hospitals would want to implement mandatory vaccination is a bit contradictory. This contrast could be caused by the fact that hospital managements think the ethical concerns outweigh the health benefits, or the hospitals do not want to take away the freedom of choice from their medical staff. The lack of legal permission for mandatory vaccination will probably also play a big role in this matter. However, the ethical discussion about this subject is increasingly being raised*.* Such mandatory vaccination programs are more likely to reach a high vaccination rate (>90%) and these rates will probably be sustained for a long period of time [12,15,16]. Van Delden et al. showed the pros and cons of mandatory vaccination, and concluded that the advantages of mandatory vaccination outweigh the burdens and risks [12]. However, in the Netherlands as in many European countries there is no legal basis for implementing mandatory vaccination in health care workers yet. Ethical discussions are currently ongoing but preferably vaccine uptake should be raised voluntarily.

## Conclusion

In conclusion, agreement of hospital management with positive statements about influenza vaccination was not associated with the uptake. Economic investments were low and more economic investments were related with a higher vaccine uptake. Reasons for the higher uptake should be explored further preferably by more qualitative methods. When vaccine uptake remains too low, only a minority of the general hospital administrators would consider implementing a mandatory vaccination program, and such a policy may take some time and efforts before is generally accepted.

## Competing interests

All authors declare that they have no competing interests.

## Authors’ contributions

EH initiated and supervised the study. JCFK and ARP acquired the data and contributed to the design. JRD drafted and wrote the main part of the manuscript and conducted the statistical analysis. All authors helped draft the manuscript, and read and approved the final manuscript.

## Pre-publication history

The pre-publication history for this paper can be accessed here:

http://www.biomedcentral.com/1471-2458/12/1101/prepub
